# Single and combined use of red cell distribution width, mean platelet volume, and cancer antigen 125 for differential diagnosis of ovarian cancer and benign ovarian tumors

**DOI:** 10.1186/s13048-018-0382-3

**Published:** 2018-01-22

**Authors:** Yuan-yuan Qin, Yang-yang Wu, Xiao-ying Xian, Jin-qiu Qin, Zhan-feng Lai, Lin Liao, Fa-quan Lin

**Affiliations:** Department of Clinical Laboratory, The First Affiliated Hospital of Guangxi Medical University, Guangxi Zhuang Autonomous Region, Nanning, China

**Keywords:** Red cell distribution width, Mean platelet volume, Cancer antigen 125, Ovarian cancer

## Abstract

**Background:**

Cancer is widely believed to result from chronic inflammation, and red cell distribution width (RDW) and mean platelet volume (MPV) are considered as inflammatory markers for cancer. We investigated the values of RDW, MPV, and cancer antigen 125 (CA125), alone or in combination, for distinguishing between ovarian cancer and benign ovarian tumors.

**Methods:**

The study included 326 patients with ovarian cancer, 290 patients with benign ovarian tumors, and 162 control subjects. Hematologic tests were performed at initial diagnosis.

**Results:**

RDW was increased and MPV was decreased in the ovarian cancer group compared with the control and benign ovarian tumor groups. RDW was positively correlated and MPV was negatively correlated with cancer stage. Area under the curve (AUC) analysis for ovarian cancer versus benign ovarian tumors revealed that the specificity and sensitivity were increased for the combination of MPV and CA125 compared with either marker alone, and the specificity was increased for the combination of RDW and CA125, compared with either alone. The AUCs for RDW plus CA125 and MPV plus CA125 were significantly larger than for any of the markers alone.

**Conclusions:**

In conclusion, combinations of the markers RDW, MPV, and CA125 may improve the differential diagnosis of ovarian cancer and benign ovarian tumors.

## Background

Ovarian cancer is one of the most common malignant tumors of the gynecological system, with the highest mortality rate of all gynecological tumors [1]. The ovaries are located in the pelvic cavity and are thus relatively concealed, in addition to which early ovarian cancer lacks any obvious clinical manifestations and diagnostic methods, making it difficult to diagnose early and to distinguish from benign ovarian tumors. More than 70% of patients with ovarian cancer are therefore initially diagnosed at an advanced stage, and the 5-year survival rate is only 30%. Ovarian cancer thus presents a serious threat to women’s health [2]. Cancer antigen 125 (CA125) is a clinical ovarian tumor marker, but its sensitivity is relatively low and other markers are therefore needed to allow discrimination between early ovarian cancer and benign ovarian tumors.

The red cell volume distribution width (RDW) is a quantitative parameter indicating the size of the red blood cells. RDW reflects red cell volume heterogeneity and is usually measured as part of the whole blood cell count. Several studies have suggested that a high RDW may be closely related to endometrial, ovarian, and liver cancers [3–5]. Furthermore, activated platelets are involved in cancer progression and metastases [6, 7]. Mean platelet volume (MPV) is a marker of activated platelets and has been associated with gastric, thyroid, and ovarian cancers [8–10]. Both RDW and MPV have recently been studied in various diseases. The aim of the current study was to investigate the roles of RDW, MPV, and CA125, either alone or in combination, for distinguishing between ovarian cancer and benign ovarian tumors.

## Methods

### Patients

We performed a retrospective study in patients diagnosed with ovarian cancer at the First Affiliated Hospital of Guangxi Medical University, China, from January 2015 to May 2017. Patients who had undergone complete surgical resection with a histologically confirmed diagnosis of ovarian cancer, and who were untreated before diagnosis were included in the study. Patients with diabetes mellitus, cardiovascular disease, kidney disease, blood disease, acute inflammation, anemia, recent iron therapy, venous thrombosis for > 6 months, and recent blood transfusions (within the last 3 months) were excluded. Patients with ovarian cancer were classified into groups according to cancer stage, in accordance with the standards established by the International Federation of Gynecology and Obstetrics in 2000 [11]. Patients diagnosed with benign ovarian tumors (mature ovarian teratoma, simple ovarian cyst, ovarian endometriosis) in our hospital during the same time period comprised the benign ovarian tumor group, and healthy subjects were selected as the control group. There were no marked differences in age among the three groups. This study was approved by the ethics committee of the First Affiliated Hospital of Guangxi Medical University, China.

### Method

Venous blood (2 mL) was collected from each patient in the morning and placed in EDTA-K2 anticoagulation tubes and drying tubes. Whole blood cell parameters were determined using a Beckman Coulter LH 780 hematology analyzer (Beckman Coulter, Brea, CA, USA). The white blood cell count, absolute neutrophil count, absolute lymphocyte count, absolute monocyte count, hemoglobin concentration (Hb), blood platelet count (PLT), MPV, platelet distribution width (PDW), and RDW were obtained directly by the hematology analyzer. Serum CA125 levels were detected using a Roche E6000 analyzer (Roche Diagnostics, Basel, Switzerland).

### Statistical analysis

All data were analyzed using SPSS 20.0 software (IBM Corp., Armonk, NY). Continuous variables are expressed as mean ± standard deviation or median (interquartile range), and categorical variables are expressed as numbers and percentages. Differences in baseline characteristics among the three groups were analyzed by one-way ANOVA. Differences in relevant indicators between two groups were compared using Tukey’s test. Correlations between RDW and PDW and cancer stage in patients with ovarian cancer were analyzed by Spearman’s correlation. Sensitivity and specificity were defined by receiver-operating characteristic curves, and differences in the area under the curve (AUC) were detected using MedCalc version 15.0. A *P* value of < 0.05 was considered statistically significant.

## Results

A total of 326 patients with ovarian cancer (range 27–81 years) were included in this study. According to the grading standards, 118 patients (36.2%) had stage I cancer, 65 (19.9%) had stage II, 83 (25.5%) had stage III, and 60 (18.4%) had stage IV. A further 290 patients with benign ovarian tumors (range 20–71 years) and 162 healthy control subjects (range 22–62 years) were also included in the study. White blood cell count, absolute neutrophil count, absolute lymphocyte count, absolute monocyte count, Hb, PLT, MPV, PDW, RDW, and CA125 differed significantly among the three groups (Table 1).

**Table 1:** Laboratory characteristics of the participants

Variables	ovarian cancer	benign ovarian tumors	controls	*P*-value
Number	326	290	162	
Age(years)	43.15 ± 11.59	43.18 ± 9.25	44.13 ± 6.90	0.544
W; (10^9^/L)	8.28 ± 4.01^a^	6.69 ± 2.25	6.33 ± 1.49^c^	<0.001
N; (10^9^/L)	6.11 ± 4.04^a^	4.12 ± 2.01	3.70 ± 1.12^c^	<0.001
L; (10^9^/L)	1.52 ± 0.68^a^	1.97 ± 0.67	2.07 ± 0.72^c^	<0.001
Mo; (10^9^/L)	0.49 ± 0.01^a^	0.45 ± 0.15	0.44 ± 0.15^c^	0.001
Hb; (g/L)	106.38 ± 19.53^a^	124.64 ± 13.13	128.14 ± 6.47^c^	<0.001
PLT; (10^12^/L)	216.50 ± 130.92^a^	239.68 ± 88.08	248.18 ± 64.8^c^	0.001
MPV; (fl)	8.19 ± 0.86^a^	9.31 ± 0.91^b^	9.64 ± 0.63^c^	<0.001
PDW;(%)	0.16 ± 0.01^a^	0.16 ± 0.02^b^	0.16 ± 0.01^c^	<0.001
RDW;(%)	0.16 ± 0.02^a^	0.14 ± 0.01^b^	0.13 ± 0.01^c^	<0.001
CA125; U/mL	68.85(32.20–385.05)^a^	23.15(14.43–37.76)	5.94(4.04–13.94)^c^	<0.001

Data are expressed as mean ± standard deviation or median (interquartile range)

W, white blood cell count; N, absolute neutrophil count; L, absolute lymphocyte count; Mo, absolute monocyte count; Hb, hemoglobin; PLT, blood platelet count; MPV, mean platelet volume; PDW, platelet distribution width; RDW, red cell distribution width; CA125, cancer antigen 125

*P* values were calculated by one-way ANOVA tests

^a^Indicates a significant difference (*P* < 0.05) between ovarian cancer and benign ovarian tumors (Tukey’s test)

^b^Indicates a significant difference (*P* < 0.05) between benign ovarian tumors and controls (Tukey’s test)

^c^Indicates a significant difference (P < 0.05) between ovarian cancer and controls (Tukey’s test)

10^9^,10^^^9; 10^12^,10^^^12

**Fig. 1:**
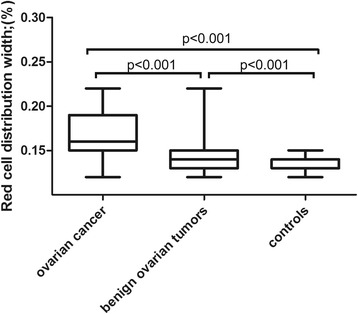
Red cell distribution width in patients with ovarian cancer or benign ovarian tumors and in healthy controls

**Fig. 2:**
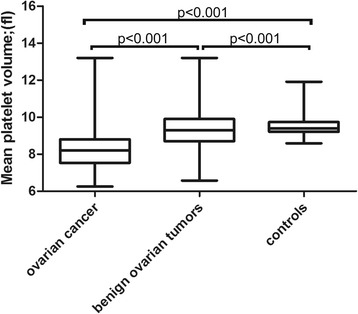
Mean platelet volume in patients with ovarian cancer or benign ovarian tumors and in healthy controls

RDW and MPV levels in patients with ovarian cancer or benign ovarian tumors and in healthy individuals are shown in Figs. 1 and 2. RDW was higher in the ovarian cancer group compared with both the control and benign ovarian tumor groups (cancer vs. benign ovarian tumors, *P* < 0.001; cancer vs. control, P < 0.001; benign ovarian tumors vs. control, P < 0.001; Tukey’s test). However, MPV was lower in the ovarian cancer group compared with the control and benign ovarian tumor groups (cancer vs. benign ovarian tumor, P < 0.001; cancer vs. control, *P* < 0.001; benign ovarian tumor vs. control, *P* < 0.001; Tukey’s test).

**Fig. 3:**
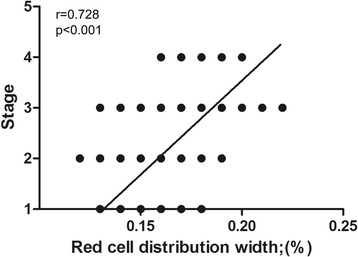
Correlation between red cell distribution width and cancer stage

**Fig. 4:**
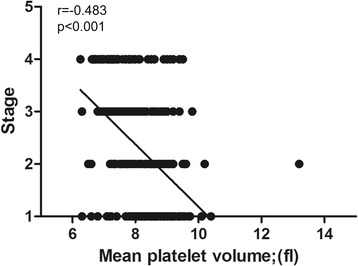
Correlation between mean platelet volume and cancer stage

Correlations between cancer stage and RDW and MPV in patients with ovarian cancer are shown in Figs. 3 and 4. Correlation analysis demonstrated that RDW was positively correlated and MPV was negatively correlated with cancer stage.

**Fig. 5:**
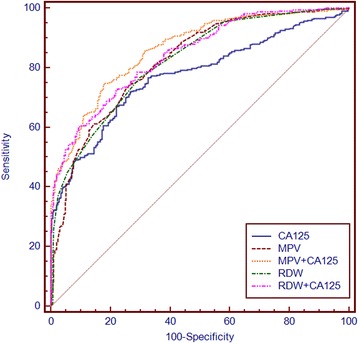
Receiver-operator characteristics curves for RDW, MPV, and CA125 alone or combined showing sensitivity and 100-specificity for the differential diagnosis of ovarian cancer versus benign ovarian tumors. RDW, red cell distribution width; MPV, mean platelet volume; CA125, cancer antigen 125

Receiver-operating characteristic analysis was used to assess the AUCs for single and combined biomarkers (Table 2). RDW and MPV had high sensitivities for distinguishing between ovarian cancer and benign ovarian tumors (76.70% and 74.20%, respectively), while MPV and CA125 had high specificities (73.8% and 73.4%, respectively). The specificity and sensitivity increased when MPV and CA125 were combined, and the specificity increased when RDW and CA125 were combined. Moreover, the combination of RDW plus CA125 manifested a significantly larger AUC (0.844, 0.813–0.872) compared with RDW and CA125 alone (*P* = 0.013 and *P* < 0.001, respectively), and the combination of MPV and CA125 manifested a significantly larger AUC (0.862, 0.833–0.889) compared with MPV and CA125 alone (both P < 0.001) (Fig. 5).

**Table 2:** Receiver operating characteristic curve analyses showing the utilities of single and combined markers for differentiating between ovarian cancer and benign ovarian tumors

Markers	Sensitivity	Specificity	+PV	-PV	AUC
RDW;(%)	76.70	70.30	74.40	72.90	0.823(0.791–0.852)
MPV;fl	74.20	73.80	76.10	71.80	0.823(0.790–0.852)
CA125;U/mL	71.80	73.40	75.20	69.80	0.772(0.737–0.804)
RDW + CA125	60.74	90.34	87.60	67.20	0.844(0.813–0.872)
MPV + CA125	74.85	82.07	82.40	74.40	0.862(0.833–0.889)

+PV, positive predictive value; -PV, negative predictive value; AUC, area under curve; RDW, red cell distribution width; MPV, mean platelet volume; CA125, cancer antigen 125

## Discussion

Early diagnosis and treatment of ovarian cancer can improve the 5-year survival rate to > 90%, compared with < 50% in patients with a late diagnosis [12]. The main diagnostic methods for ovarian cancer are currently mainly gynecological examination, tumor marker detection, imaging, cytology, and histology, though all these tests have some limitations. The identification of early ovarian cancer markers is thus of great importance in terms of improving the diagnosis, treatment efficacy, and prognosis of patients. The results of the current study showed that RDW levels were significantly higher and MPV levels were significantly lower in patients with ovarian cancer compared with patients with benign ovarian tumors and healthy controls. Furthermore, various combinations of RDW, MPV, and CA125 were valuable for diagnosing ovarian cancer and distinguishing it from benign ovarian tumors.

Cancer is widely believed to be the result of chronic inflammation [4]. Inflammatory cytokines have been shown to play a role in inhibiting the stimulatory effect of erythropoietin on bone marrow erythrocyte stem cells, anti-apoptosis, and cell maturation, thus causing more immature red blood cells to be released into the peripheral blood circulation, thereby increasing the heterogeneity of peripheral red blood cells and RDW [13]. Elevated RDW may also be associated with an increased rate of ineffective hematopoiesis caused by chronic inflammation. Hunziker et al. demonstrated that the inflammatory response and oxidative stress could affect erythropoiesis, and alter blood cell membrane deformability and erythrocyte half-life, thereby increasing RDW [14]. Ovarian cancer has certain characteristics of a chronic inflammatory disease, and levels of many cytokines, including tumor necrosis factor-a, interleukin (IL)-1, and IL-17, are unbalanced in patients with ovarian cancer,cytokines may affect the production, apoptosis, size, and fragility of red blood cells through different pathways [15]. Neote et al. confirmed that many types of inflammatory factor receptors were expressed on the surface of red blood cells, and suggested that red blood cells were involved in the inflammatory process [16]. Patients with advanced ovarian cancer often have impaired gastrointestinal and immune functions, which may result in deficiencies of iron, folic acid, vitamin B12, and other red blood cell metabolites, various degrees of anemia; and an increased RDW.

MPV is a marker of platelet function and activation that can be determined easily during complete blood counts, with no additional cost. Various studies have identified MPV as a useful indicator in some inflammatory diseases, and it has been associated with disease activity and severity of inflammation [17]. Incebiyik et al. reported low MPV values in pelvic inflammatory disease, and emphasized its diagnostic value [18]. Reduced MPV levels have also been implicated in non-small cell lung cancer, multiple myeloma, and severe primary dysmenorrhea [19–21]. However, the mechanism responsible for the low MPV in patients with ovarian cancer remains unclear. Inflammation and coagulation have been shown to mediate the immune response, which in turn plays an important role in tumor development, invasion, and metastasis [22]. Some factors, such as IL-1, IL-6, and granulocyte colony-stimulating factor can indirectly increase platelet production [23, 24], and considerable evidence suggests that IL-6 promotes tumorigenesis by regulating apoptosis, survival, angiogenesis, metastasis, and metabolism [25]. In addition, megakaryopoiesis and subsequent thrombopoiesis in cancer may be stimulated by granulocyte colony-stimulating factor and macrophage colony-stimulating factor, which can be secreted by tumor cells [26]. Both basic and clinical studies have found a link between malignancy and platelet abnormalities [27]. Platelets play a key role in regulating inflammation. Released inflammatory mediators increase platelet activation, leading to a subsequent change in MPV. The consumption of platelets at the inflammation site may be responsible for the decrease in MPV. Furthermore, the inflammatory environment can impair megakaryopoiesis, in turn causing the release of small platelets from the bone marrow [28].

CA125 is a commonly used biochemical marker for ovarian cancer diagnosis. However, CA125 levels may also be increased in patients with some benign gynecological lesions, such as endometriosis and pelvic inflammatory disease, making it prone to false positive results [29]. The current study showed that, compared with RDW, MPV, and CA125 alone, MPV combined with CA125 had higher sensitivity and specificity for distinguishing between ovarian cancer and benign ovarian tumors, and RDW combined with CA125 had higher specificity. The combinations of CA125 with RDW or MPV manifested significantly larger AUCs compared with RDW, MPV, and CA125 alone, suggesting that using these combined markers may improve the early detection of ovarian cancer and its differential diagnosis from benign ovarian tumors.

This study had some limitations. This was a relatively small retrospective study in patients with ovarian cancer, and the small sample size prevented us from drawing any firm conclusions about the correlations between RDW, MPV, and ovarian cancer. Further, large-scale prospective studies are therefore needed to confirm these results. Furthermore, this study only included Chinese participants, and the results therefore cannot be generalized to other ethnic groups. Nevertheless, to the best of our knowledge, this study provides the first evidence for the combined use of RDW, MPV, and CA125 for discriminating between ovarian cancer and benign ovarian tumors.

## Conclusions

Early diagnosis of ovarian cancer and its distinction from benign ovarian tumors are essential for improving its prognosis. CA-125 alone is insufficient for this purpose, but combinations of RDW, MPV, and CA125 may facilitate the early detection and differential diagnosis of ovarian cancer compared with benign ovarian tumors.

## Abbreviations

CA125: Cancer antigen 125
Hb: Hemoglobin
L: Absolute lymphocyte count
Mo: Absolute monocyte count
MPV: Mean platelet volume
N: Absolute neutrophil count
PDW: Platelet distribution width
PLT: Blood platelet count
RDW: Red cell distribution width
W: White blood cell count
